# Developing and Applying the BE-FAIR Equity Framework to a Population Health Predictive Model: A Retrospective Observational Cohort Study

**DOI:** 10.1007/s11606-025-09462-1

**Published:** 2025-03-14

**Authors:** Reshma Gupta, Mayu Sasaki, Sandra L. Taylor, Sili Fan, Jeffrey S. Hoch, Yi Zhang, Matthew Crase, Dan Tancredi, Jason Y. Adams, Hendry Ton

**Affiliations:** 1 Office of Population Health and Accountable Care, University of California (UC) Davis Health, Sacramento, CA USA; 2 Department of Medicine, UC Davis, Sacramento, USA; 3 Department of Public Health Sciences, UC Davis, Davis, USA; 4 Center for Healthcare Policy and Research, UC Davis, Sacramento, USA; 5 Department of Pediatrics, UC Davis, Sacramento, USA; 6 IT Data Center of Excellence, UC Davis, Sacramento, USA; 7 Center for Health Equity, Diversity, and Inclusion, UC Davis, Sacramento, USA; 8 Department of Psychiatry and Behavioral Sciences, UC Davis, Sacramento, USA; 9 Division of Health Policy and Management, UC Davis, Davis, USA

**Keywords:** predictive models, population health, bias, equity framework, fairness

## Abstract

**Background:**

Population health programs rely on healthcare predictive models to allocate resources, yet models can perpetuate biases that exacerbate health disparities among marginalized communities.

**Objective:**

We developed the Bias-reduction and Equity Framework for Assessing, Implementing, and Redesigning (BE-FAIR) healthcare predictive models, an applied framework tested within a large health system using a population health predictive model, aiming to minimize bias and enhance equity.

**Design:**

Retrospective cohort study conducted at an academic medical center. Data collected from September 30, 2020, to October 1, 2022, were analyzed to assess bias resulting from model use.

**Participants:**

Primary care or payer-attributed patients at the medical center identified through electronic health records and claims data. Participants were stratified by race-ethnicity, gender, and social vulnerability defined by the Healthy Places Index (HPI).

**Intervention:**

BE-FAIR implementation involved steps such as an anti-racism lens application, de-siloed team structure, historical intervention review, disaggregated data analysis, and calibration evaluation.

**Main Measures:**

The primary outcome was the calibration and discrimination of the model across different demographic groups, measured by logistic regression and area under the receiver operating characteristic curve (AUROC).

**Results:**

The study population consisted of 114,311 individuals with a mean age of 43.4 years (SD 24.0 years), 55.4% female, and 59.5% white/Caucasian. Calibration differed by race-ethnicity and HPI with significantly lower predicted probabilities of hospitalization for African Americans (0.129±0.051, *p*=0.016), Hispanics (0.133±0.047, *p*=0.004), AAPI (0.120±0.051,* p*=0.018), and multi-race (0.245±0.087, *p*=0.005) relative to white/Caucasians and for individuals in low HPI areas (0 – 25%, 0.178±0.042, *p*<0.001; 25 – 50%, 0.129±0.044, *p*=0.003). AUROC values varied among demographic groups.

**Conclusions:**

The BE-FAIR framework offers a practical approach to address bias in healthcare predictive models, guiding model development, and implementation. By identifying and mitigating biases, BE-FAIR enhances the fairness and equity of healthcare delivery, particularly for minoritized groups, paving the way for more inclusive and effective population health strategies.

**Supplementary Information:**

The online version contains supplementary material available at 10.1007/s11606-025-09462-1.

## BACKGROUND

For patients with complex health conditions, population health programs use healthcare predictive models to determine which patients are most in need of scarce resources.^1–3^ Despite increasing use, models are not consistently evaluated for bias that may exacerbate health disparities, defined as systematic and avoidable health differences that adversely affect socially disadvantaged groups.^4,5^ If patient groups are underrepresented or their experiences misunderstood during predictive model development and implementation, they risk being left out of population health outreach, particularly minoritized communities who already experience health inequities resulting from racism, cultural bias, and other forms of social disadvantage.^4–13^

Predictive model bias can arise at multiple points in model development and implementation, from problem formulation, study design, data selection, modeling decisions, and evaluation. Areas of potential bias include underrepresentation of patient groups (e.g., small sample size,^14,15^ missing or non-representative data, access, and trust),^14–19^ use of outcome measures that inappropriately characterize minoritized groups and propagate historical inequities (e.g., trust, access, underinsurance, undertreatment),^4,20,21^ and insufficient evaluation of bias in model performance overtime.^22^ Even models that do not show overt bias in retrospective analysis can negatively impact patients due to bias during implementation.

Biased predictive models pose risks to minoritized patients and are a focus of scientific research and federal policy.^14,23–25^ Multiple frameworks have emerged to guide healthcare institutions, model developers, and industry.^26–28^ Despite these tools, it remains challenging to develop models that minimize bias and mitigate residual bias during implementation. Two major literature and policy gaps include guidance on strategies to reduce data processing bias and impacts of model use on equity. Further, frameworks remain focused on theoretical aspects without sufficient guidance on implementation. To create equitable population health programs, health systems must address bias that is perpetuated from outside and within the health system.

UC Davis Health (UCDH) developed BE-FAIR (i.e., a Bias-reduction and Equity Framework for Assessing, Implementing, and Redesigning healthcare predictive models). While previous approaches lack real-world application and specificity to health system implementation,^26–28^ BE-FAIR is an applied framework for responsible artificial intelligence (AI) tested in a large health system. This framework incorporates bias reducing data processing strategies with racial equity impact assessments^29^ to examine how minoritized groups may be affected by models. The approach uses open-source tools for others to implement locally. We describe a case study of a population health predictive model where we used BE-FAIR to improve fairness, defined as reducing bias and systemic discrimination in model design, implementation, and evaluation.^30,31^

## METHODS

This retrospective cohort study was exempt from review and informed consent by the institutional review board at the University of California Davis (IRB # 2062965-1).

### Study Population and Data Source

UCDH encompasses two hospitals of 646 beds, 70 ambulatory clinics, 2200 physicians seeing over a million visits annually, and 160,000 patients across 33 counties. UCDH previously introduced primary care managers assigned to each clinic to coordinate care and respond to patients’ acute clinical decline. To enhance patient care, UCDH developed a machine learning (ML) predictive model to facilitate identifying patients who may benefit from care management services (i.e., future 12-month risk of hospitalization or emergency department (ED) visits). This model used electronic health record (EHR) and claims data to generate input features, ascertain patient outcomes, and define patient subgroups.

Using the BE-FAIR framework, we assessed bias by studying differences in future 12-month predicted versus actual unplanned hospitalizations and ED visits between patients of different race-ethnicity, gender, and location. Location data were used to define social vulnerability by the Area Deprivation Index (ADI)^32,33^ and California-specific Healthy Places Index (HPI).^33^ Data was collected from 9/30/2020 to 10/1/2021 with the outcome, unplanned admissions, or ER visits, collected from 10/1/2021 to 10/1/2022. The team used HPI data as it aligned with other state and county-based outreach efforts. (See Appendix: Table A1, metric definitions.) We analyzed all metrics to be complete and valid based on chart reviews of randomly sampled patients and encounters.

**Table 1:** Bias-reduction and Equity Framework for Assessing, Implementing, and Redesigning (BE-FAIR) Healthcare Predictive Models

Step	Common pitfalls	Specific ACTIONS
1. Apply an anti-racism lens to address bias from the start. [Aligns AHRQ/NIMHHD Principle #1, 5]* • An anti-racism approach posits all projects are at risk for perpetuating biases that pre-exist in health care. These biases can be mitigated if meaningful health equity goals are explicitly prioritized at the project inception	Health equity goals are identified as an afterthought allowing bias to set in including race-based assumptions (i.e., differences in health outcomes are due to biological differences between racial groups), leading to strategies that ignore contributing biases inherent in the health system (e.g., non-standardized data collection protocols leading to patterns of missing data), and making it more difficult to pivot to bias mitigating strategies	• Focus on addressing inequities caused by racism and other forms of social disadvantage rather than perpetuating assumptions that the inequities inherently arise from an individual’s race (aka race-based assumptions) • Identify components of the project plan based on race-based assumptions and mitigate them • Develop specific components of the project plan designed to identify and address the impact of racism and inequities in health including human input into workflows using healthcare predictive models and addressing potential to exacerbate the “digital divide”
2. Engage partners that have insights into the impacted communities alongside those with technical expertise. [Aligns AHRQ/ NIMHHD Principle #3, 4]* • When community perspectives are meaningfully incorporated with technical expertise, project decisions are better informed, interpretation of data become richer, and interventions are more relevant. This step ensures that underrepresented groups are explicitly considered, and builds collective knowledge of the needs, perspectives, and experiences of these groups	Design decisions are made with considerations of only dominant groups, leading to missed opportunities to address inequities of marginalized groups, or even unintentionally worsening these inequities Community engagement is under resourced and/or underprioritized, leading to harmful interactions with communities, eroding trust in current and future efforts.	• Partner with health equity researchers, community leaders, and/or DEI offices throughout the project—these partners can inform the process through their work and relationship with impacted communities but should also flag when direct community input is needed. • Solicit input directly from impacted community via focus groups, surveys, and/or key informant interviews on project design, data interpretation, and project implementation. • Utilize best practices in community engagement (such as NIH Principles of Community Engagement)
3. Review the history of health system interactions with locally underserved communities. [Aligns AHRQ/NIMHHD Principle #3, 4]*	Lack of understanding community history and experiences can lead to erroneous interpretation of the data, less relevant and less effective interventions, and diminished community trust	• Identify which groups are historically and/or locally underserved by the health system • Review known literature on disparities experienced by these groups • Review how model design decisions may potentially improve, exacerbate, or newly create inequities for these groups • Put in place safeguards including methods for assessing these inequities
4. Disaggregate and assess the baseline data for training model across racial, cultural, and socioeconomic demographics • Disaggregation of data is essential for understanding how interventions impact different racial, cultural, and socioeconomic groups	Programs that do not disaggregate data are unable to detect and understand differences between groups	• Disaggregate data across races, genders, ages, abilities, and socioeconomic backgrounds, and underserved and marginalized groups. • Incorporate social determinants of health indicators • Improve workflows and data collection to mitigate lack of representation of data to include in training model • Work with other institutions and compliance offices or health exchanges to share data when numbers from underrepresented groups are too small
5. Select technical features with hypothesized causal relationships to the desired outcome. Use technical factors that include race and social determinants particularly given racism’s pervasive yet often unrecognized influence on outcomes • While race itself should be considered as a factor, avoid automatic conclusions about race being a causal factor, as racism may be the cause	Failure to assess for differences in outcomes by race and other social and demographic factors potentially obscures the impact of bias on outcomes	• Explicitly address health inequity associated with the outcome chosen and across focused groups or health conditions • Define approach to missing data considering differential effects of imputation and complete case strategies • Summarize technical features by outcome and group including extent of missing values
6. Transparently evaluate predictive models for differential performance among groups or exclusion of patients when models are used to mitigate bias. [Aligns AHRQ/ NIMHHD Principle #4]*	Failure to assess model performance or data quality differences among groups and to integrate mitigation actions in program implementation can perpetuate health disparities	• Carefully consider which performance metrics are most important clinically and are most important for achieving outcome equity • Assess models for differential performance (calibration and discrimination) among different groups • Engage health equity subject matter experts, community collaborators, and patients to better understand the context and potential cause(s) for differential outcomes to characterize them as true health disparities rather than health differences unrelated to social disadvantage
7. Utilize CLAS Standards, cultural humility, and trustworthiness strategies to inform intervention design and implementation. [Aligns AHRQ/NIMHHD Principle #3]* Once inequities are detected, health equity and community engagement perspectives should inform the design and implementation of the intervention	Teams designing or interventions to address health inequities in underrepresented groups may design culturally incongruent interventions or make missteps in service delivery that diminish trust or exacerbate the inequities	• Identify and address how biases arise during implementation.\ • Partner with health equity/DEI leads and community partners to utilize the cultural humility training for project to educate about the history, context, impact of racism/inequity • Focus improvement strategies and trainings on addressing bias arising from patient, care team, institutional, and structural factors rather than patient factors alone • Make information available for patients and communities about when the predictive model is used in their care and how the model performs for their sociodemographic group • Protect patient autonomy and privacy in predictive model development, implementation, and monitoring
8. Institute inclusive and equitable continuous improvement. [Aligns AHRQ/ NIMHHD Principle #2]* • Sustained efforts are often needed to address health inequities	Programs that do not incorporate a health equity lens into its continuous improvement process may not be able to keep pace with changing community needs and demographics, sustain efforts to build and maintain trust with impacted communities, or maintain their health equity improvements	• Monitor for performance and impact on minoritized populations after model implementation • Adjust project goals to keep pace with changing community needs and demographics • Focus on formal evaluation for bias, continuous monitoring, and formal oversight for model drift considering that over time the patient group may change
9. Address underlying structural inequities through advocacy, education, and strategies • Individual projects may not have the scope or resources to address the pervasive root causes of health inequities. Projects help to characterize these structural inequities for health system leaders who can implement system level changes. Project teams can also educate and advocate for equitable policy changes at a societal level	Lack of coordinated systemic and policy strategies to address underlying issues contributes to the persistence of health inequities	• Assess the potential for and how the process can help make changes within the organization to eliminate institutional and structural racism • Consider advocacy for issues that would benefit from wider policy change • Utilize a system-wide anchor institution model to address social determinants of health

*AHRQ/NIMHHD Principles: (1) promoting health and healthcare equity throughout healthcare predictive model life cycles, (2) ensuring predictive models are transparent and explainable, (3) authentically engaging patients and communities throughout the life cycle of predictive model development and earning trustworthiness, (4) identifying predictive model fairness issues and trade-offs, and (5) establishing accountability for equity and fairness in outcomes from predictive models^26^

### Framework and Approach

UCDH created a multispecialty leadership team to develop a framework to assess, mitigate, and monitor for bias in system-wide predictive models including population health, equity, and information technology experts. Patient input was elicited for aspects of development. The team met bimonthly over 2 years to create and implement Bias-reduction and Equity Framework for Assessing, Implementing, and Redesigning (BE-FAIR) healthcare predictive models, which includes steps to evaluate and mitigate bias in model development and implementation (Table 1).

The team applied BE-FAIR to the population health predictive model. This model provides care managers with predicted probabilities for future 12-month hospitalizations or ED visits for individual patients. Patients above a threshold percentile of risk (i.e., 60% or greater defined due to outreach staff bandwidth limitations) are identified, and, with primary care clinician guidance, determined if they may benefit from program enrollment. If appropriate, staff proactively contact patients, provide needs assessments, and begin pre-defined care management workflows.

Framework implementation as intended for the population health predictive model:

*Steps 1–2: Apply an anti-racism lens to proactively identify and address bias and race-based assumptions from the start. Meaningfully engage partners that have insights into the perspectives and experiences of the impacted communities.* The team created a charter prioritizing health inequity across all initiative phases that could implement across primary care clinics uniformly. The charter outlined a mission statement aimed to transparently evaluate for bias, defined a diverse planning team, defined model development and evaluation phases, and proactively identified relevant systematic, structural, and interpersonal risks of bias for each phase. For example, the planning team members represented *diverse* race/ethnicities, community representation, disciplines, clinical specialties, and content expertise. During metric selection, the team assessed for missing data to address potential under coverage bias (i.e., inadequate representation within data), while during the patient engagement phase, the risk of non-response and self-selection bias (i.e., only easy-to-reach patients engage) led to registry-based outreach workflows. Through charter development, there was early recognition that patient care decisions would not be dictated by the predictive model alone but rather final decisions were made by human clinical judgement. The team elicited patient feedback to understand perspectives about the consequences of using the model and how best to communicate about use of computer algorithms in care. They also prioritized routinely examining systems-related root causes (e.g., missing data, outcome prevalence, healthcare access, trust) rather than attributing causes to patient race.

*Step 3: Review the history of health system interventions among locally underserved communities.*The team reviewed the literature for known risks of bias in population predictive models, including bias caused by missing data, selection of model input features, and outcomes. They also evaluated the local history of mistrust between the health system and local minoritized communities with health equity experts, many of whom grew up in or aware of community perspectives. These interviews identified concern that some metrics were hard to capture among certain groups that may face barriers (e.g., underrepresented communities with lower rates of ambulatory visits or transportation barriers) and have missing data so the team suggested improved raw data collection workflows.^34–37^ Some patients also noted competing priorities that made answering the phone difficult during outreach so the team improved workflows to reach out at different times thus allowing patients to engage when they could.

*Step 4–5: Disaggregate baseline data for training models across racial, cultural, and socioeconomic demographics. Select technical features with clear causal relationship to the desired outcome, avoiding blind use of classifications such as race, and establish equitable outcomes across different groups as the desired goal.* The team assessed baseline data for the availability and quality of data disaggregated by race-ethnicity, gender, and HPI. The team reviewed each candidate model feature and patterns of missing data within patient groups. Discussion points considered how use of differentially missing data may result in underrepresentation of patient groups and choice of metrics including geolocation (e.g., HPI, ADI) and healthcare utilization patterns over time. The outcome measure was predetermined as there was an operational need to reduce unplanned hospitalizations and ED visits. The team conducted additional analyses to assess each outcome alone and combined after assessing differences by race-ethnicity, gender, and HPI. Ultimately, the 31-feature predictive model was developed from 215 candidate features hypothesized to correlate with the outcome.

*Step 6: Transparently develop and evaluate healthcare predictive models for differential performance among populations or exclusion of patients when models are used to mitigate bias.* The team evaluated calibration and discrimination to identify differential performance of the model across groups at different levels of predicted risk to help identify where the model increased risk of bias.

*Step 7: Utilize Culturally and Linguistically Appropriate Services (CLAS), cultural humility, and trustworthiness strategies to inform intervention design and implementation:*Care management outreach staff were trained on how structural racism and other forms of social disadvantage impact patient engagement and ways to address patient concerns about bias and mistreatment that foster trustworthiness.^38^ Decisions for patient care (e.g., patient outreach) were not dictated by the predictive model alone; rather, final outreach decisions were made by human clinical judgment (i.e., care management staff assess appropriateness for enrollment based on chart review and patient conversations). The team also exercised transparency by creating an electronic health record smart phrase to explain to patients that a model was used to identify them for outreach.

*Step 8: Institute inclusive and equitable continuous improvement:* The team performed a formal evaluation of bias (see the “Statistical Analysis” section). The continuous monitoring and oversight for model drift over time (i.e., assessed model performance statistics and population demographic changes) was measured and reported to an institution-wide, multi-disciplinary oversight committee to ensure transparency.

*Step 9: Address underlying structural inequities through advocacy, education, and anchor strategies:* The team initiated an education program to teach health system leaders on how to utilize the BE-FAIR framework to mitigate bias in other areas. The team also advocated for more culturally informed approaches to engage populations identified in this project and developed regional workforce and procurement anchor strategies to improve the social determinants of health in under resourced neighborhoods.

### Statistical Analysis

As part of Step 8, to institute inclusive and equitable continuous improvement, the team assessed model bias in a retrospective cohort using calibration (i.e., comparing predicted risk to observed outcome rates) and discrimination metrics^39,40^ across pre-specified patient groups. To evaluate differential calibration intercepts and slopes, the team used logistic regression to model observed events over a 12-month period versus the predicted probability from the model (logit transformed) with inclusion of a group variable (race- ethnicity, HPI, or gender) and their interaction with predicted probability. Significant main effects for the group variable indicate differential calibration intercepts and a significant interaction indicates differential calibration slopes by group. We report log odds ratios, 95% confidence limits, and *p*-values for these analyses. For discrimination, we estimated the area under the receiver operating characteristic curve (AUROC) and compared 95% confidence limits across groups. We further calculated sensitivity, specificity, positive predictive value, and negative predictive value at a decision threshold of 60, selected *a priori* based on operational constraints. We used standard confusion matrix metrics instead of any single derived fairness metric because they can be directly evaluated for fairness or can be converted to other fairness metrics. Although our model predicted the combined outcome of hospitalization or ER visit, we also evaluated these two outcomes separately. Encounters with missing values for risk scores, gender, race-ethnicity, or HPI were dropped (*N* = 31,470, 21.6%) after analysis showed no substantive differences between those with or without missing values. All statistical analyses were conducted using R Statistical Computing Software Version 4.2.1.

## RESULTS

### Population Description

The study participants were diverse with 40.5% identifying as a race-ethnicity other than white/Caucasian. The prevalence of hospitalizations was approximately twice that of ED visits (hospitalization 4.6%; ED visit 2.2%). African Americans experience higher rates of both event types relative to other groups (Table 2). Prevalences of both event types were highest in the lowest HPI quartiles and declined with the highest HPI (Table 2).

**Table 2:** Number of Patients by Race-Ethnicity, Gender, Age, HPI Quartiles, and Prevalence of ED Visits, Unplanned Hospitalizations for These Groups in the Evaluation Set

	Analytical dataset^a^ (*N* = 114,311)	ED visit prevalence* (*N* = 2525)	Hospitalization prevalence* (*N* = 5271)
Age (years), mean (SD)	43.4 (24.0)	-	-
Gender^b^			
Female	63,345 (55.4%)^b^	1491 (2.4%)	3192 (5.0%)
Male	50,966 (44.6%)	1035 (2.0%)	2079 (4.1%)
Race/ethnicity^b^			
White/Caucasian	68,061 (59.5%)	1367 (2%)	2989 (4.4%)
AIAN^¥^	415 (0.4%)	8 (1.9%)	25 (6%)
Black	6207 (5.4%)	322 (5.2%)	550 (8.9%)
Multi-racial	4180 (3.7%)	96 (2.3%)	182 (4.4%)
AAPI^¥^	15,172 (13.3%)	243 (1.6%)	546 (3.6%)
Hispanic	15,806 (13.8%)	393 (2.5%)	773 (4.9%)
Other	4470 (3.9%)	97 (2.2%)	206 (4.6%)
HPI^±^ quartiles^b^			
HPI 0–25%	28675 (25.1%)	1051 (3.7%)	1984 (6.9%)
HPI 25–50%	28677 (25.1%)	599 (2.1%)	1344 (4.7%)
HPI 50–75%	28541 (25%)	475 (1.7%)	1097 (3.8%)
HPI 75–100%	28418 (24.9%)	401 (1.4%)	846 (3%)

^a^Full dataset contained 145,781 patients. After dropping patients with missing values, the analytical dataset contained 114,311 patients. Percentages are reported based on the analytical dataset (*N*=114,311)

^b^Based on the full dataset (*N*=145,781), the number and percentage of missing values were gender (64 [0.04%]), race/ethnicity (14,175 [9.7%]), and HPI (10419 [7.1%])

*Counts and percentages are prevalences of ED visits or hospitalizations for each group. For example, 1367 (2%) white/Caucasians had an ED visit

^¥^*AIAN*, American Indian or Alaskan Native; *AAPI*, Asian American or Pacific Islander

^±^*HPI*, Healthy Places Index (healthier community conditions have higher HPIs)

### Model Calibration and Discrimination

**Figure 1:**
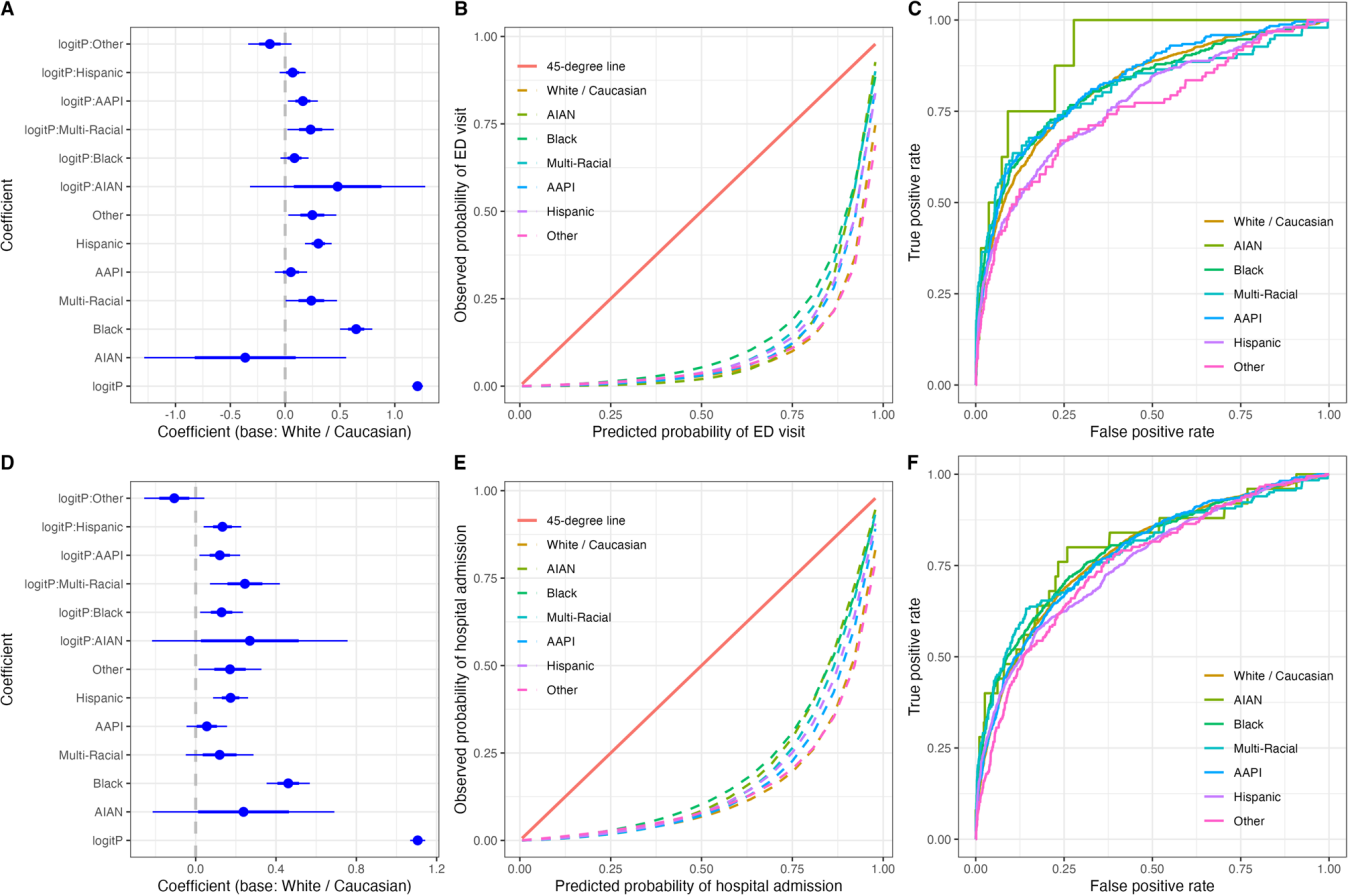
Calibration assessment and ROC curves of model performance by race/ethnicity. Log odds ratios and 95% confidence intervals from the logistic regression model evaluating calibration in predicting **A)** ED visits and **D)** unplanned hospitalizations by race/ethnicity. Calibration curves by race/ethnicity for predicting **B)** ED visits and **E)** unplanned hospitalizations. Receiver operating characteristic curves of model performance adjusting for race/ethnicities for **C)** ED visits and **F)** unplanned hospitalizations.

There was evidence of differential calibration by race-ethnicity for both ED visits and hospitalizations. Because a balanced dataset (equal numbers of patients with and without an emergency room visit or unplanned hospitalization) was used to train the model, predicted event probabilities were substantially higher than observed probabilities given the lower event prevalence rate. Nevertheless, the team observed hospitalization calibration intercepts and slopes were significantly higher for African Americans and Hispanics compared to white/Caucasians, indicating slightly better calibration between predicted and actual utilization for African Americans and Hispanics compared to white/Caucasians (Fig. 1; Appendix: Table A2). Observed ED use also was significantly higher for AAPI and multi-race relative to white/Caucasians at a given predicted use (Fig. 1; Appendix: Table A3).

**Table 3:** Model Performance Metrics [95% Confidence Limits] for ED Visit and Unplanned Hospitalization for Race and HPI by Groups for a Decision Threshold of 60

Attribute	AUROC*	Sens*	Spec*	PPV*	NPV*
**Emergency department visits**					
Race/ethnicity					
White	0.817 [0.804, 0.829]	0.495 [0.468, 0.521]	0.923 [0.921, 0.925]	0.116 [0.108, 0.124]	0.989 [0.988, 0.99]
AIAN^¥^	0.909 [0.835, 0.983]	0.75 [0.349, 0.968]	0.907 [0.874, 0.933]	0.136 [0.052, 0.274]	0.995 [0.981, 0.999]
Black	0.818 [0.791, 0.845]	0.599 [0.544, 0.653]	0.89 [0.882, 0.898]	0.23 [0.202, 0.26]	0.976 [0.972, 0.98]
Multi^¥^	0.809 [0.754, 0.865]	0.438 [0.336, 0.543]	0.95 [0.943, 0.957]	0.171 [0.126, 0.224]	0.986 [0.982, 0.99]
AAPI^¥^	0.829 [0.801, 0.857]	0.412 [0.349, 0.476]	0.953 [0.95, 0.956]	0.125 [0.103, 0.15]	0.99 [0.988, 0.992]
Hisp^¥^	0.773 [0.747, 0.799]	0.402 [0.353, 0.452]	0.94 [0.937, 0.944]	0.147 [0.126, 0.169]	0.984 [0.982, 0.986]
Other	0.752 [0.696, 0.808]	0.381 [0.285, 0.486]	0.943 [0.935, 0.949]	0.128 [0.092, 0.173]	0.986 [0.982, 0.989]
Healthy Places Index quartiles					
0–25	0.801 [0.786, 0.816]	0.548 [0.517, 0.578]	0.893 [0.89, 0.897]	0.164 [0.152, 0.176]	0.981 [0.979, 0.983]
25–50	0.809 [0.79, 0.828]	0.484 [0.443, 0.525]	0.924 [0.921, 0.927]	0.119 [0.107, 0.133]	0.988 [0.987, 0.989]
50–75	0.793 [0.771, 0.816]	0.427 [0.382, 0.473]	0.94 [0.937, 0.943]	0.108 [0.094, 0.122]	0.99 [0.989, 0.991]
75–100	0.8 [0.775, 0.826]	0.357 [0.31, 0.406]	0.959 [0.957, 0.962]	0.112 [0.095, 0.13]	0.99 [0.989, 0.992]
**Unplanned hospitalizations**					
Race-ethnicity					
White	0.781 [0.772, 0.79]	0.392 [0.375, 0.41]	0.928 [0.926, 0.93]	0.201 [0.191, 0.212]	0.971 [0.969, 0.972]
AIAN^¥^	0.798 [0.694, 0.902]	0.48 [0.278, 0.687]	0.918 [0.886, 0.943]	0.273 [0.15, 0.428]	0.965 [0.941, 0.981]
Black	0.792 [0.771, 0.814]	0.511 [0.468, 0.553]	0.902 [0.893, 0.909]	0.335 [0.303, 0.368]	0.95 [0.944, 0.956]
Multi^¥^	0.786 [0.746, 0.825]	0.368 [0.298, 0.443]	0.955 [0.948, 0.961]	0.272 [0.218, 0.333]	0.971 [0.965, 0.976]
AAPI^¥^	0.781 [0.761, 0.802]	0.3 [0.262, 0.341]	0.956 [0.953, 0.96]	0.205 [0.177, 0.234]	0.973 [0.971, 0.976]
Hisp^¥^	0.757 [0.739, 0.776]	0.351 [0.317, 0.385]	0.946 [0.943, 0.95]	0.252 [0.226, 0.279]	0.966 [0.963, 0.969]
Other	0.754 [0.719, 0.79]	0.282 [0.221, 0.348]	0.946 [0.939, 0.953]	0.201 [0.157, 0.252]	0.965 [0.959, 0.97]
Healthy Places Index quartiles					
0–25	0.769 [0.757, 0.781]	0.462 [0.44, 0.484]	0.902 [0.899, 0.906]	0.26 [0.246, 0.275]	0.958 [0.955, 0.96]
25–50	0.78 [0.766, 0.793]	0.386 [0.36, 0.413]	0.93 [0.927, 0.933]	0.213 [0.197, 0.23]	0.969 [0.966, 0.971]
50–75	0.768 [0.752, 0.783]	0.328 [0.3, 0.357]	0.944 [0.942, 0.947]	0.191 [0.173, 0.209]	0.972 [0.97, 0.974]
75–100	0.765 [0.747, 0.783]	0.271 [0.241, 0.302]	0.962 [0.96, 0.964]	0.179 [0.158, 0.201]	0.977 [0.975, 0.979]

Sample sizes for each group are shown in Table 2

*AUROC, area under the receiver operating characteristic curve; *Sens*, sensitivity; *Spec*, specificity; *PPV*, positive predictive value; *NPV*, negative predictive value

^¥^*AIAN*, American Indian or Alaskan Native; *AAPI*, Asian-American or Pacific Islander; *Hisp*, Hispanic; *Multi*, multi-racial

**Figure 2:**
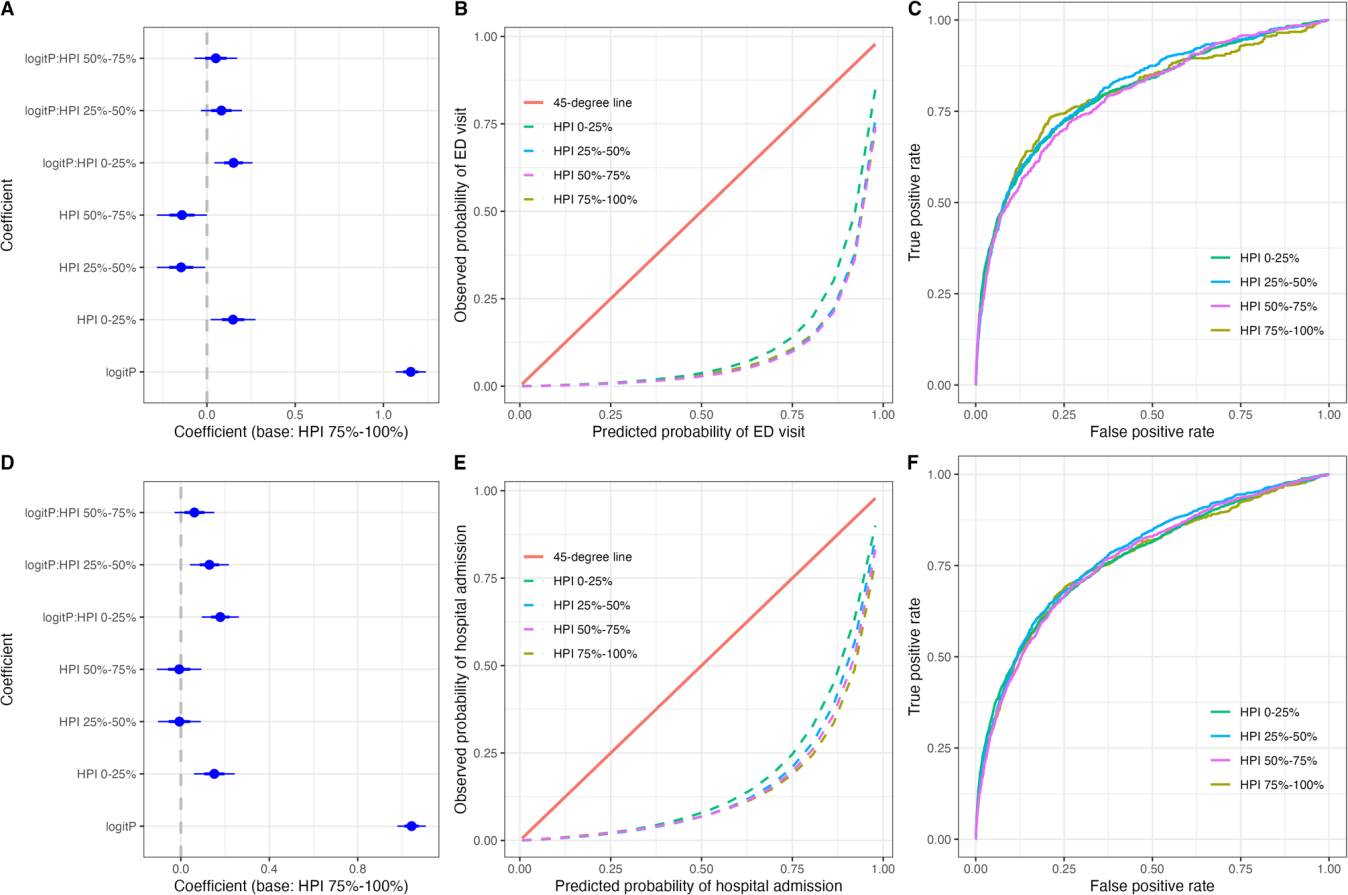
Calibration assessment and ROC curves of model performance by Healthy Places Index quartiles. Log odds ratios and 95% confidence intervals from the logistic regression model evaluating calibration in predicting **A)** ED visits and **D)** unplanned hospitalizations by HPI quartiles. Calibration curves by race/ethnicity for predicting **B)** ED visits and **E)** unplanned hospitalizations. Receiver operating characteristic curves of model performance adjusting for HPI quartile for **C)** ED visits and **F)** unplanned hospitalizations.

Predicted probabilities for ED visits and hospitalizations were significantly lower than observed use for the lowest HPI quartile group (0–25%, signifying individuals living in the least healthy environments) relative to the highest (Fig. 2; Appendix: Tables A4 and A5). Differential calibration was not detected between genders for either outcome (Appendix: Tables A6 and A7; Figure A1).

The AUROC for ED visits was lowest and below 0.8 for the Hispanic and Other race group; AUROC was highest for AIAN although this may reflect the small sample size of this group. Test sensitivity for a decision threshold of 60 also varied among race/ethnicity group with sensitivity lowest among Hispanic and the Other race group and highest among African Americans (Table 3). AUROC values were similar for ED visits across HPI quartiles but sensitivity was notably higher for the lowest HPI quartile (0–25%) (Table 3).

For unplanned hospitalizations, AUROC estimates were similar across races (Table 3). Sensitivity was highest for African Americans but low for AAPI and Other race groups. For HPI, model discrimination was similar across the quartiles although like ED visits, sensitivity was highest for the HPI 0–25% quartile. Discrimination metrics for gender are provided in the Appendix: Tables A8 and A9.

We evaluated missing data by estimating the percent missing for each feature overall and by race. We found ADI (5.6%) and HPI (7.4%) had the highest missing data, which was due to not having addresses on file for these patients. The percentage of missing data for all other features was <5%. Patients within the unknown race category had 17% missing data and this population had clinical conditions that make it hard for patients to answer survey questions.

## DISCUSSION

The BE-FAIR approach made the systematic evaluation of bias feasible and improved fairness in implementation and oversight for a real-world population health predictive model. Application of the framework helped to develop a carefully curated dataset that improved representation of the community. BE-FAIR’s comparative assessment of population-specific model performance and incorporation of the calibration evaluation led to identification of outreach thresholds based on predicted risk levels to improve the fairness of population health outreach. These analytic methods can be easily applied in other health systems to assess for bias. While predictive model development is resource intensive, these evaluations are feasible for both internally or vendor-developed models with a part-time statistical analyst.

Using the framework helped the evaluation team to identify historical bias (e.g., non-response, self-selection, and under coverage biases). Other bias mitigation strategies identified included standardizing data collection workflows to better capture data for patients with systematic patterns of missing data. For example, staff received training and performance transparency about their data collection practices for patients with conditions affecting memory. Outreach teams also received training to incorporate cultural humility and modify outreach practices such as incorporating multiple outreach attempts at different times of the month to increase potential engagement for patients with competing priorities. Operations teams maintained the threshold for patient outreach (i.e., 60% or greater) as it both reduced potential bias among some groups while maximizing outreach staff bandwidth. The BE-FAIR framework also guided alignment of a distinct and uniquely skilled team of predictive analytics, equity, and health system leaders. Previously published frameworks have been limited in bridging these often-siloed areas required for real-world implementation and ensuring input from and responsibility to patients.^26,27^

The BE-FAIR study is one of the few predictive model evaluations in population health with real-world application that incorporates operational and technical strategies.^26,28^ Strategies to reduce data processing bias and a racial equity impact assessment helped to identify how underrepresented,^4–12^ minoritized groups may be affected by model use. The predictive model underpredicted the probability of hospitalizations and ED visits for specific subgroups compared to white/Caucasians as seen in prior literature.^41,42^ The underprediction among multi-race and AAPI groups is due to their small, fixed size and heterogeneity of these populations. The model underestimated predicted probabilities for people living in areas of the lowest HPI quartile, underscoring the complexity of predicting utilization in low-income patients due to unaccounted factors related to SDOH, complex health needs, health literacy, and provider bias.^43,44^

The landmark 2019 Obermeyer study uncovering predictive model bias fueled more evaluations of bias in the use of models,^4^ yet no one bias mitigation strategy has been identified as best practice.^26^ Many studies propose high-level non-technical guidelines for unbiased model development,^9,11,22,45–47^ while others provide highly specific technical guidance for only a portion of the model development cycle.^48,49^ A more comprehensive framework by the Agency for Healthcare Research and Quality (AHRQ) and the National Institute for Minority Health and Health Disparities (NIMHHD) identified five guiding principles to address during predictive model development. They include promoting health and healthcare equity throughout model life cycles, ensuring models are transparent and explainable, authentically engaging patients and communities throughout the life cycle of model development and earning trustworthiness, identifying model fairness issues and trade-offs, and establishing accountability for equity and fairness in outcomes from models.^27^ Most studies have not followed all five of these guiding principles during predictive model development,^26^ and many studies lack demonstration of the practical, real-world application of the model in the clinical setting. The BE-FAIR approach addresses the AHRQ and NIMHHD identified principles in partnership with social science, technical, statistical, and clinical experts (Table 1, aligned principles).^26^

While predictive models have the potential to democratize care access and improve health outcomes, rapidly evolving health technologies enter clinical and operational use at a speed incongruous with the thoughtful approach required to address deeply entrenched societal and health inequities. This dilemma poses risk to minoritized groups if we fail to develop responsible and just methods to define, develop, evaluate, implement, and monitor the impacts of emerging models. BE-FAIR offers a beginning-to-end framework to identify opportunities to mitigate potential bias for minoritized patient groups.

Even with the practical application of an anti-bias framework, this evaluation is limited by generalizability and health system structural barriers. As a single-center study, the findings may not generalize to health systems lacking resources in model development and those lacking leadership commitment to equity and responsible artificial intelligence. This application of BE-FAIR also does not address generative artificial intelligence models where model development and evaluation standards are still in their infancy. Nonetheless, BE-FAIR attempts to leverage universal approaches to bias evaluation and address common pitfalls of artificial intelligence tools that could be tested among broader applications in the future. Other limitations include known health system structural barriers for minority groups in accessing care or providing data that may be factors in underestimating and overestimating outcomes.

## CONCLUSION

BE-FAIR serves as a bridge, though not exhaustive, between three pillars to reduce healthcare predictive model bias through community engagement, data science methods, and implementation science. While prediction models can perform well among a diverse population, this evaluation identified differences in performance among underrepresented groups and mitigation strategies for potential bias when applied within health systems.^50^

## Supplementary Information

Below is the link to the electronic supplementary material.

Supplementary file1 (DOCX 457 KB)
