# Supplementary material for: Developing and Applying the BE-FAIR Equity Framework to a Population Health Predictive Model: A Retrospective Observational Cohort Study
Source: J Gen Intern Med. 2025 Mar 14;40(11):2537–47. doi: 10.1007/s11606-025-09462-1 (PMC12405130; doi:10.1007/s11606-025-09462-1)

**Supplementary Information**

***APPENDIX**

**Table A1: Metric Definitions**

| **Evaluation Features** | **Definition** |
| --- | --- |
| Race-Ethnicity | American Indian or Alaska Native; Black or African American; Multi-racial; Asian, Native Hawaiian or Other Pacific Islander; Latinx/Hispanic; Other; and White/Caucasian. |
| Gender | Male, Female, Other or Unknown |
| Healthy Places Index (HPI) | HPI was defined by quartile-membership where lower quartiles represent greater social and health vulnerability, eligible values were whole numbers between 1 and 10.^33^ |
| Unplanned Hospitalizations | A predictable/preventable admission as fulfilling the criteria:  A non-trauma, non-newborn delivery in the future 12 months or  Two or more admissions of any kind in the future 12 months |
| Unplanned ED Visits | A predictable/preventable ED visit as fulfilling the criteria:  Visit with a principal diagnosis in a CMS Hierarchical Condition Category in the future 12 months, or  Three or more ED-visits of any kind in the future 12 months |
| **Population Health Algorithm Features** | **Description** |
| Area Deprivation Index - National | Area deprivation index national percentile median ranking for the patient's zip code: https://www.neighborhoodatlas.medicine.wisc.edu/ |
| Area Deprivation Index - State | Area deprivation index state decile median ranking for the patient's zip code: https://www.neighborhoodatlas.medicine.wisc.edu/ |
| California Healthy Places Index | The Healthy Places Index (HPI) is a data and mapping tool that measures the health and well-being of communities by assessing social and environmental factors: https://www.healthyplacesindex.org/ |
| Cardiologist Referral Ever | Yes/No, Has the patient ever had a cardiologist referral? |
| Cardiologist Visit Ever | Yes/No, Has the patient ever completed a cardiologist visit? |
| Cardiologist Visit in last 24 months | How many cardiologist visits in the past 24 months has the patient completed? |
| Pulmonologist Visit Ever | Yes/No, Has the patient ever completed a Pulmonologist visit |
| MD Encounters- 12 Months | The number of appointments, in-person office visits or qualifying virtual encounters with a physician in the last 12 months |
| Non-MD Encounters- 12 Months | The number of appointments, in-person office visits or qualifying virtual encounters, with a non-physician in the last 12 months |
| MD Encounters- 6 Months | Yes/No has the patient had an in-person office visit or qualifying virtual encounter with a physician in the 6 months prior to the snapshot date. |
| Non-MD Encounters- 6 Months | Yes/No has the patient had an in-person office visit or qualifying virtual encounter with a non-physician in the 6 months prior to the snapshot date. |
| Unique MDs- 12 Months | The number of distinct physicians that the patient has had one or more in-person office visit or qualifying virtual encounter within the last 12 months |
| Unique Non-MDs- 12 Months | The number of distinct non-physicians that the patient has had one or more, in-person office visit or qualifying virtual encounter within the last 12 months |
| Unique Continuity Specialists | Number of distinct specialties in which the patient has had 2 or more visits with the same specialty physician in the last 18 months |
| Health Plan- Medicare | Yes/No, patient is a member of a Medicare health plan |
| Health Plan- Medicare Shared Savings | Yes/No, patient is a member of CMS Medicare Shared Savings Program (MSSP) |
| Patient Age | The patient's age, as an integer value |
| Ejection Fraction | Codified for low score of echocardiogram |
| Total Count of Chronic Conditions | The number of Chronic Condition Warehouse medical conditions documented for the patient. Categories found here: https://www2.ccwdata.org/web/guest/condition-categories |
| Individual Chronic Conditions- separate metrics for each condition | Meets the CMS Chronic Condition Warehouse criteria for Anemia, CKD, COPD, Diabetes, Heart Failure, Hypertension, Hyperlipidemia, Ischemic Heart Disease, Rheumatoid Arthritis or Osteoarthritis |
| Creatinine Level | Codified based on the patient’s Creatinine level |
| Glucose Level | Codified based on the patient’s urine glucose level |
| Bronchodilator Use | Yes/No Bronchodilator |
| Active Filled Meds | The number of distinct outpatient medications filled at the hospital pharmacy or identified via third party claim within the measurement period. |
| Hospital admissions | Number of hospital admissions in the last 12 months |
| ED Only Visits | Number of Emergency Department Visits by patient in the last 12 months where patient was not admitted from the ED but discharged |
| No Hospitalization or ED Visit | Yes/No, patients have had no hospital admissions or ED visits in the prior year |
| Exposure Proportion | The number of the previous 6 quarters with at least one ED only visit or admission |
| UCDH Multi-Visit Patient | Indicates that the patient has had >= 4 ED-only visits in the last 12 months, or >= 4 inpatient admissions in the last 12 months |
| Chronic Advanced Illness | Institution definition of advanced illness for kidney failure, liver failure, heart failure, cancer, and high dose opiates |
| Chronic Advancing Illness | Institution definition of developing advanced illness including diabetes, kidney failure, livery failure, heart failure, cancer, and high dose opiates |

| **Table A2:** Model coefficients for the logistic regression evaluating model calibration for an unplanned hospitalization by race. Independent variables were the logit transformation of the predicted probability from the predictive model (logitP), race and their interaction. | | | | | | | | | | | | |
| --- | --- | --- | --- | --- | --- | --- | --- | --- | --- | --- | --- | --- |
|  | Estimate | | Std. Error | | z value | | Pr(>\|z\|) | | 2.5 % | | 97.5 % | |
| (Intercept) | -2.628 | | 0.020 | | -133.842 | | 0.000 | | -2.666 | | -2.589 | |
| logitP | 1.106 | | 0.019 | | 58.905 | | 0.000 | | 1.069 | | 1.142 | |
| AIAN | 0.238 | | 0.226 | | 1.053 | | 0.293 | | -0.235 | | 0.658 | |
| Black | 0.461 | | 0.054 | | 8.567 | | 0.000 | | 0.354 | | 0.565 | |
| Multi-Racial | 0.119 | | 0.084 | | 1.416 | | 0.157 | | -0.049 | | 0.282 | |
| AAPI | 0.055 | | 0.050 | | 1.096 | | 0.273 | | -0.044 | | 0.154 | |
| Hispanic | 0.174 | | 0.044 | | 3.983 | | 0.000 | | 0.088 | | 0.259 | |
| Other | 0.171 | | 0.078 | | 2.184 | | 0.029 | | 0.015 | | 0.322 | |
| logitP:AIAN | 0.270 | | 0.243 | | 1.107 | | 0.268 | | -0.189 | | 0.774 | |
| logitP:Black | 0.129 | | 0.053 | | 2.415 | | 0.016 | | 0.025 | | 0.235 | |
| logitP:Multi-Racial | 0.245 | | 0.087 | | 2.816 | | 0.005 | | 0.077 | | 0.419 | |
| logitP:AAPI | 0.120 | | 0.051 | | 2.374 | | 0.018 | | 0.021 | | 0.220 | |
| logitP:Hispanic | 0.133 | | 0.047 | | 2.844 | | 0.004 | | 0.042 | | 0.226 | |
| logitP:Other | -0.106 | | 0.075 | | -1.422 | | 0.155 | | -0.253 | | 0.041 | |
| **Table A3:** Model coefficients for the logistic regression evaluating model calibration for an emergency dependent visit by race. Independent variables were the logit transformation of the predicted probability from the predictive model (logitP), race and their interaction. | | | | | | | | | | | | |
|  | | Estimate | | Std. Error | | z value | | Pr(>\|z\|) | | 2.5 % | | 97.5 % |
| (Intercept) | | -3.524 | | 0.029 | | -120.503 | | 0.000 | | -3.582 | | -3.467 |
| logitP | | 1.207 | | 0.025 | | 48.071 | | 0.000 | | 1.158 | | 1.256 |
| AIAN | | -0.364 | | 0.460 | | -0.791 | | 0.429 | | -1.442 | | 0.414 |
| Black | | 0.647 | | 0.074 | | 8.777 | | 0.000 | | 0.500 | | 0.790 |
| Multi-Racial | | 0.239 | | 0.117 | | 2.039 | | 0.041 | | 0.002 | | 0.462 |
| AAPI | | 0.053 | | 0.074 | | 0.719 | | 0.472 | | -0.094 | | 0.195 |
| Hispanic | | 0.303 | | 0.061 | | 4.973 | | 0.000 | | 0.182 | | 0.421 |
| Other | | 0.248 | | 0.109 | | 2.265 | | 0.024 | | 0.027 | | 0.457 |
| logitP:AIAN | | 0.479 | | 0.400 | | 1.198 | | 0.231 | | -0.252 | | 1.358 |
| logitP:Black | | 0.085 | | 0.064 | | 1.330 | | 0.184 | | -0.039 | | 0.212 |
| logitP:Multi-Racial | | 0.232 | | 0.106 | | 2.196 | | 0.028 | | 0.028 | | 0.444 |
| logitP:AAPI | | 0.162 | | 0.068 | | 2.372 | | 0.018 | | 0.029 | | 0.296 |
| logitP:Hispanic | | 0.068 | | 0.059 | | 1.154 | | 0.248 | | -0.047 | | 0.185 |
| logitP:Other | | -0.139 | | 0.099 | | -1.410 | | 0.159 | | -0.333 | | 0.055 |

| **Table A4:** Model coefficients for the logistic regression evaluating model calibration for an unplanned hospitalization by Healthy Places Index (HPI) quartile. Independent variables were the logit transformation of the predicted probability from the predictive model (logitP), HPI and their interaction. | | | | | | |
| --- | --- | --- | --- | --- | --- | --- |
|  | Estimate | Std. Error | z value | Pr(>\|z\|) | 2.5 % | 97.5 % |
| (Intercept) | -2.611 | 0.0384 | -67.943 | < 0.001 | -2.687 | -2.540 |
| logitP | 1.044 | 0.032 | 32.372 | < 0.001 | 0.981 | 1.1070 |
| HPI 0-25% | 0.151 | 0.046 | 3.278 | 0.001 | 0.061 | 0.242 |
| HPI 25%-50% | -0.007 | 0.048 | -0.140 | 0.888 | -0.101 | 0.088 |
| HPI 50%-75% | -0.008 | 0.050 | -0.154 | 0.877 | -0.106 | 0.091 |
| logitP:HPI 0-25% | 0.178 | 0.0420 | 4.245 | < 0.001 | 0.096 | 0.261 |
| logitP:HPI 25%-50% | 0.129 | 0.044 | 2.937 | 0.003 | 0.0430 | 0.215 |
| logitP:HPI 50%-75% | 0.061 | 0.045 | 1.354 | 0.175 | -0.027 | 0.149 |

| **Table A5:** Model coefficients for the logistic regression evaluating model calibration for an emergency department visit by Healthy Places Index (HPI) quartile. Independent variables were the logit transformation of the predicted probability from the predictive model (logitP), HPI and their interaction. | | | | | | |
| --- | --- | --- | --- | --- | --- | --- |
|  | Estimate | Std. Error | z value | Pr(>\|z\|) | 2.5 % | 97.5% |
| (Intercept) | -3.396 | 0.052 | -64.982 | 0.000 | -3.500 | -3.295 |
| logitP | 1.155 | 0.043 | 27.022 | 0.000 | 1.071 | 1.239 |
| HPI 0-25% | 0.148 | 0.064 | 2.324 | 0.020 | 0.024 | 0.273 |
| HPI 25%-50% | -0.146 | 0.068 | -2.138 | 0.032 | -0.280 | -0.012 |
| HPI 50%-75% | -0.142 | 0.071 | -1.997 | 0.046 | -0.280 | -0.002 |
| logitP:HPI 0-25% | 0.151 | 0.054 | 2.790 | 0.005 | 0.045 | 0.256 |
| logitP:HPI 25%-50% | 0.082 | 0.058 | 1.410 | 0.158 | -0.032 | 0.196 |
| logitP:HPI 50%-75% | 0.050 | 0.061 | 0.831 | 0.406 | -0.068 | 0.169 |

| **Table A6**: Model coefficients for the logistic regression evaluating model calibration for an unplanned hospitalization visit by gender. Independent variables were the logit transformation of the predicted probability from the predictive model (logitP), gender and their interaction. | | | | | | |
| --- | --- | --- | --- | --- | --- | --- |
|  | Estimate | Std. Error | z value | Pr(>\|z\|) | 2.5 % | 97.5 % |
| (Intercept) | -2.652 | 0.024 | -111.782 | 0.000 | -2.699 | -2.606 |
| logitP | 1.185 | 0.022 | 52.901 | 0.000 | 1.141 | 1.229 |
| FEMALE | 0.175 | 0.030 | 5.752 | 0.000 | 0.116 | 0.235 |
| logitP:FEMALE | -0.053 | 0.030 | -1.787 | 0.074 | -0.111 | 0.005 |

| **Table A7:** Model coefficients for the logistic regression evaluating model calibration for an emergency department visit by gender. Independent variables were the logit transformation of the predicted probability from the predictive model (logitP), gender and their interaction. | | | | | | |
| --- | --- | --- | --- | --- | --- | --- |
|  | Estimate | Std. Error | z value | Pr(>\|z\|) | 2.5 % | 97.5 % |
| (Intercept) | -3.463 | 0.034 | -102.343 | 0.000 | -3.530 | -3.397 |
| logitP | 1.263 | 0.029 | 43.999 | 0.000 | 1.207 | 1.319 |
| FEMALE | 0.110 | 0.044 | 2.507 | 0.012 | 0.024 | 0.196 |
| logitP:FEMALE | -0.023 | 0.038 | -0.588 | 0.556 | -0.098 | 0.053 |

**Table A8**: Performance metrics [95% Confidence Limits] for unplanned hospitalization by gender for a decision threshold of 60

| Attribute | AUROC ^*^ | Sens ^*^ | Spec ^*^ | PPV ^*^ | NPV ^*^ |
| --- | --- | --- | --- | --- | --- |
| Female | 0.77 [0.761, 0.779] | 0.366 [0.349, 0.383] | 0.931 [0.929, 0.933] | 0.22 [0.209, 0.232] | 0.965 [0.964, 0.967] |
| Male | 0.794 [0.783, 0.805] | 0.412 [0.391, 0.434] | 0.939 [0.937, 0.941] | 0.224 [0.211, 0.238] | 0.974 [0.973, 0.975] |
| ^*^ AUROC = area under the receiver operating characteristic curve, Sens = sensitivity, Spec = specificity, PPV = positive predictive value, NPV = negative predictive value. | | | | | |

| **Table A9:** Model performance metrics [95% confidence limits] for emergency department visits for gender groups for a decision threshold of 60. | | | | | |
| --- | --- | --- | --- | --- | --- |
| Attribute | AUROC^*^ | Sens^*^ | Spec^*^ | PPV^*^ | NPV^*^ |
| GENDER |  |  |  |  |  |
| Female | 0.802 [0.79, 0.802] | 0.466 [0.441, 0.492] | 0.926 [0.923, 0.928] | 0.131 [0.122, 0.141] | 0.986 [0.985, 0.987] |
| Male | 0.827 [0.813, 0.827] | 0.5 [0.469, 0.53] | 0.934 [0.932, 0.936] | 0.135 [0.125, 0.147] | 0.989 [0.988, 0.99] |

^*^ AUROC = area under the receiver operating characteristic curve, Sens = sensitivity, Spec = specificity, PPV = positive predictive value, NPV = negative predictive value.

**Figure A1:** Calibration assessment and ROC curves of model performance by gender.


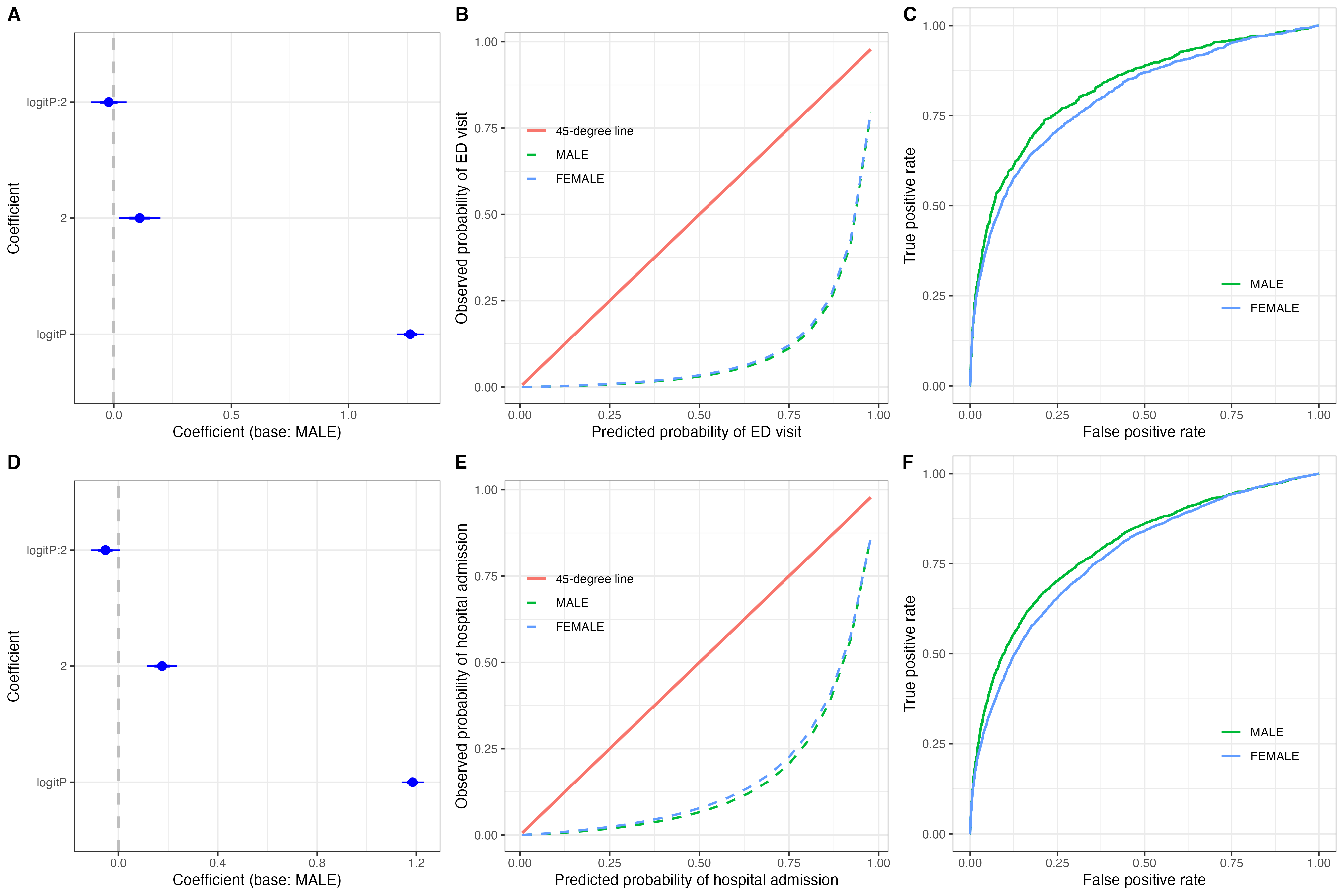

Supplement: Supplementary file 1 — Supplementary file1 (DOCX 457 KB) [file 11606_2025_9462_MOESM1_ESM.docx]
